# Exploring the Surgical Outcomes of Pancreatic Cancer Resections Performed in Low- Versus High-Volume Centers

**DOI:** 10.7759/cureus.37112

**Published:** 2023-04-04

**Authors:** Muhammad S Ghauri, Jonathan Juste, Talha Shabbir, Nicole Berry, Akshay J Reddy, Navid Farkoufar, Shabana Masood

**Affiliations:** 1 Neurosurgery, California University of Science and Medicine, Colton, USA; 2 School of Medicine, California University of Science and Medicine, Colton, USA; 3 Community and Global Health, Claremont Graduate University, Claremont, USA; 4 Medical Education, California University of Science and Medicine, Colton, USA

**Keywords:** whipple procedure, surgical mortality, surgical outcomes, pancreatic cancer, pancreatic cancer resection

## Abstract

Introduction

Pancreatic cancer resections comprise a class of complex surgical operations with a high postoperative morbidity rate. Due to the complicated nature of pancreatic resection, individuals who undergo this procedure are advised to visit a high-volume medical center that performs such pancreatic surgeries frequently. However, this specialized treatment option may not be available for uninsured patients or patients with other socioeconomic limitations that may restrict their access to these facilities. To gain a better understanding of the impact of healthcare disparities on surgical outcomes, we aimed to explore if there were significant differences in mortality rate post-pancreatic resection between high- and low-volume hospitals within San Bernardino, Riverside, Los Angeles, and Orange Counties.

Methods

We utilized the California Health and Human Services Agency (CHHS) California Hospital Inpatient Mortality Rates and Quality Ratings public dataset to compare risk-adjusted mortality rates (RA-MR) of pancreatic cancer resections procedures. We focused on procedures performed in hospitals within San Bernardino, Riverside, Los Angeles, and Orange County from 2012 to 2015. To assess post-resection outcomes in relation to hospital volume, we utilized an independent T-test (significance level was set equal to 0.05) to determine if there is a statistically significant difference in RA-MR after pancreatic resection between high- and low-volume hospitals.

Results

During the 2012-2015 study period, 57 hospitals across San Bernardino, Riverside, Orange, and Los Angeles Counties were identified to perform a total of 6,204 pancreatic resection procedures. The low-volume hospital group (N=2,539) was associated with a higher RA-MR of M=4.45 (SD=11.86). By comparison, the high-volume hospital group (N=3,665) was associated with a lower RA-MR of M=1.72 (SD=2.61).

Conclusion

Pancreatic resection surgeries performed at low-volume hospitals resulted in a significantly higher RA-MR compared to procedures done at high-volume hospitals in California.

## Introduction

Pancreatic cancer, or pancreatic ductal adenocarcinoma (PDA), ranks as the fourth leading cause of cancer-related death in Western countries, with a five-year survival rate of only 5% [1-3]. This is largely due to the insidious nature of PDA which is rarely diagnosed at a time when surgical resection is feasible. Despite ongoing research efforts, we lack markers of early detection leading to ineffective screening programs that remain unreliable even in high-risk populations [4]. Since over 80% of patients are found to have unresectable tumors at diagnosis, it is important to take advantage of tumors that are eligible to undergo resection [5]. Pancreatic cancer resections, also known as pancreaticoduodenectomies, comprise a class of complex surgical operations with a high postoperative morbidity rate of 40-50% [6-7]. Due to the complicated nature of pancreatic resection, individuals who undergo this procedure are advised to visit a high-volume (number of procedures performed within a given time period) medical center that performs such pancreatic surgeries frequently. This is because surgeons and staff at high-volume hospitals have more experience and expertise in performing these complex procedures, leading to better outcomes. Additionally, high-volume hospitals typically have better resources and access to newer technologies, which can also improve outcomes for patients [7]. However, this specialized treatment option may not be available for uninsured patients or patients with other socioeconomic limitations that may restrict their access to these facilities [8]. Many studies have shown that patients undergoing pancreatic cancer resection at low-volume hospitals have higher rates of complications and lower survival rates compared to those at high-volume hospitals. These discrepancies have been attributed to a lack of expertise and experience, inadequate resources, and centers with less specialized care [8-25]. These multifactorial disparities may also lead to higher risks of intraoperative and postoperative complications for specialized treatments like pancreatic resections. To gain a better understanding of the impact of healthcare disparities on surgical outcomes, we first aimed to explore if there were significant differences in mortality rate post-pancreatic resection between high- and low-volume hospitals within San Bernardino, Riverside, Los Angeles, and Orange Counties. With regard to pancreatic resections, a high-volume center performs more than 20 operations each year. Previous studies have attempted to elucidate risk factors that complicate the prognosis of pancreatic resection [1-25]. Here, we implement these previously identified pre-operative risk factors into a calculated risk-adjusted mortality rate (RA-MR). Understanding how these mortality rates compare between various hospitals in these counties can help healthcare professionals better address socioeconomic factors that potentially affect surgical outcomes.

## Materials and methods

Study design

We utilized the California Health and Human Services Agency (CHHS) California Hospital Inpatient Mortality Rates and Quality Ratings public dataset, a national inpatient surgical database (government-provided) comparing RA-MR of various surgical procedures. We gathered all data from pancreatic procedures performed in hospitals across California from 2012 to 2015. We included all patients that underwent pancreatic resections within lower- (San Bernardino and Riverside) and higher-income counties (Los Angeles and Orange County) to potentially identify any significant differences with RA-MR. In order to determine income status, we compared median household income compared to national averages. We implemented previously used methods to classify and group the data from each hospital as high volume if they performed >20 pancreatic resections or low volume if they performed <20 pancreatic resections during the year.  The primary objective was to assess post-resection outcomes in relation to hospital volume within four major counties in California.

Statistical analysis

Initially, we conducted a priori power calculation using G*Power (version 3.1.9.7; Heinrich Heine University Düsseldorf, Germany) to calculate the sample size needed for data collection. Next, we evaluated the homogeneity of our data distribution by using the Kolmogorov-Smirnova and Shapiro-Wilk normality tests with Lilliefors significance correction. This study utilized an independent T-test (significance level was set equal to 0.05) to determine if there is a statistically significant difference in RA-MR after pancreatic resection between high- and low-volume hospitals. All statistical analyses were performed using SPSS Statistics software V28.0.1.0 (IBM Corp. Released 2021. IBM SPSS Statistics for Windows, Version 28.0. Armonk, NY: IBM Corp).

## Results

During the 2012-2015 study period, 6,204 pancreatic resections were performed across 57 hospitals in San Bernardino (n=305), Riverside (n=219), Orange (n=1271), and Los Angeles (n=4409) Counties were identified to perform a total of 6,204 pancreatic resection procedures. The RA-MR for low- and high-volume hospitals in San Bernardino was 5.42 and 1.7, in Riverside 5.56 and 0, in Orange 2.07 and 1.29, and in Los Angeles 4.73 and 1.98 (Table 1).

**Table 1:** Mean RA-MR of all pancreatic resection cases in low- or high-volume hospitals per county RA-MR: risk-adjusted mortality rate

County	Total Cases (N=6,204)	Low Volume Mean RA-MR (# of cases)	High Volume Mean RA-MR (# of cases)
San Bernardino	305	5.42 (232)	1.7 (73)
Riverside	219	5.56 (197)	0 (22)
Orange	1,271	2.07 (457)	1.29 (814)
Los Angeles	4,409	4.73 (1,653)	1.98 (2,756)

**Figure 1:**
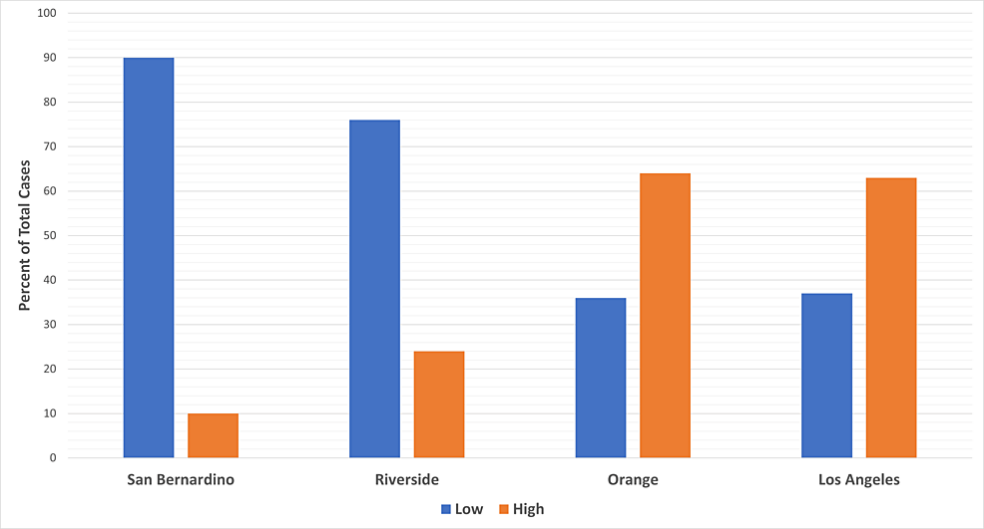
Distribution of pancreatic resection cases in low- versus high-volume centers

Interestingly, over the three-year period, the trend in the number of cases at low- versus high-volume hospitals remained fairly constant. Pancreatic resection volume differed between the four counties, with Orange and Los Angeles Counties constituting the bulk of total resections (92%). The majority of cases in these two counties were observed at high-volume centers (64% and 63%, respectively). This is in stark contrast to those of both San Bernardino (90%) and Riverside (76%) Counties, in which the majority of their resections were performed at low-volume centers (Figure 1).

**Figure 2:**
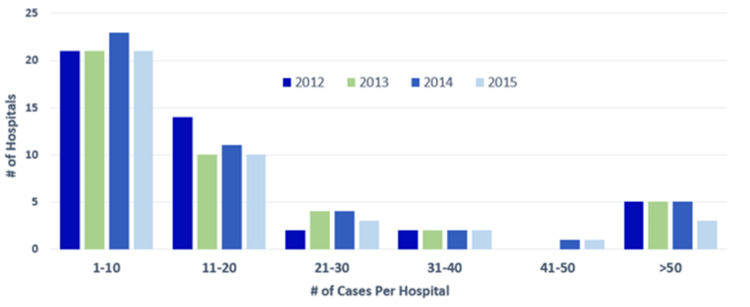
Number of hospitals with different resection volumes from 2012 to 2015

Overall, in this three-year period, we did not identify significant changes in the trend of the number of cases performed at low- versus high-volume hospitals (Figure 2).

As we hypothesized, we identified a statistically significant difference in the mean RA-MR between low- and high-volume hospital groups (p=0.001). The low-volume hospital group (N=331) was associated with a higher RA-MR of M=4.45 (SD=11.87). By comparison, the high-volume hospital group (N=87) was associated with a lower RA-MR of M=1.72 (SD=2.62) (Table 2).

**Table 2:** Independent t-test between low- and high-volume hospitals RA-MR: risk-adjusted mortality rate

Hospital Type	N	Mean RA-MR	SD	t	Sig. (two-tailed)	Mean Difference
Low volume	331	4.45	11.87	3.849	0.001	2.73
High volume	87	1.72	2.62			

## Discussion

Surgical resections for the treatment of pancreatic cancer are very complex and highly encouraged to be performed at high-volume centers. Previous studies have attributed an association between RA-MR and hospital volume [1-3,6-13]. Our research aims to supplement these studies that have investigated healthcare disparities in pancreatic cancer resection surgery. Here, we hone into four counties in Southern California included in government-provided data on state-wide inpatient surgeries. Our analysis shows that there is a statistically significant difference in the mean RA-MR between low- (M=4.45) and high-volume (M=1.72) centers (Table 2). This could be due to the fact that San Bernardino and Riverside Counties performed over 75% of resections at low-volume centers, yielding higher mortality rates. This could also be influenced by the fact that San Bernardino and Riverside Counties are considered low-income. Higher-income counties, such as Los Angeles and Orange Counties, showed the opposite trend, performing 10 times more resections with a majority done at high-volume centers and displaying nearly half the mortality rate (Table 1). Low-volume centers have worse outcomes for pancreatic cancer resection due to several reasons. Surgeons and staff at low-volume centers perform fewer procedures and have less experience, which can result in higher rates of complications and lower survival rates [22]. Low-volume centers often have limited resources, such as specialized equipment, technologies, and support staff, which can hinder the success of complex procedures like pancreatic cancer resection [24]. High-volume centers typically have multidisciplinary teams of specialists, including gastroenterologists, oncologists, and radiologists, who work together to provide specialized care for pancreatic cancer patients [25]. Low-volume centers may not have access to this level of expertise and may provide less specialized care. Therefore, patients with pancreatic cancer who undergo surgery at a high-volume center are more likely to have better outcomes compared to those at a low-volume center. These differences affect the trajectory of surgical outcomes leading to significant differences in mortality rates and possibly other complications not specifically addressed in this study.

Healthcare professionals can utilize this research to use a patient-centered approach to better plan pancreatic cancer treatment regimens. Elucidating how the spectrum of case severity influences case management can be informative in predicting which type of hospital should be performing certain procedures. For example, high-risk resections, such as the Whipple procedure, involve more complicated diagnoses and should be done solely at high-volume centers [1-3]. Our data conveys how certain counties like San Bernardino and Riverside perform fewer resections, potentially limiting the experience of surgeons in comparison with other counties (i.e., Los Angeles and Orange) leading to cases with higher mortality rates. Our study did have some limitations. Although we utilized RA-MR to control for factors that influence mortality rate, to properly evaluate surgical outcomes, it is important to identify additional patient-level variables such as length of stay, disease severity, and postoperative complications, [19-22]. Additionally, future studies should delineate exactly how socioeconomic inequity, particularly the variations in socioeconomic status (SES), county income levels, and surgeon experience, contribute to differences in the quality of the institution a patient is eligible to receive a pancreatic resection. This information could lead to better centralization of pancreatic resection surgeries allowing for more optimized distribution of care in these counties, so that “easier” resection cases are done at low-volume centers and “high-risk” complicated procedures can be performed at high-volume centers despite variation in patient SES [25].

## Conclusions

Our findings indicate that pancreatic resection surgeries performed at low-volume hospitals resulted in a significantly higher risk-adjusted mortality rate compared to procedures done at high-volume hospitals. These results highlight the importance of case management and resource allocation to ensure that these complex surgeries are performed at high-volume hospitals. Future studies are needed to delineate the factors involved in disparities leading to poorer outcomes following pancreatic resection surgeries.
